# CXCR6 expression predicts prognosis and immunotherapeutic benefit in muscle-invasive bladder cancer

**DOI:** 10.3389/fonc.2024.1498579

**Published:** 2024-11-11

**Authors:** Xiaolin Lu, Li-Ping Ge, Zhaopei Liu, Yu Zhu, Dingwei Ye, Yuan Chang

**Affiliations:** ^1^ Department of Urology, Fudan University Shanghai Cancer Center, Shanghai, China; ^2^ Department of Oncology, Shanghai Medical College, Fudan University, Shanghai, China; ^3^ Department of Breast Surgery, Fudan University Shanghai Cancer Center, Shanghai, China

**Keywords:** muscle-invasive bladder cancer, CXCR6, tumor immune microenvironment, prognostic biomarker, immunotherapy

## Abstract

**Background:**

Increasing evidence suggests that the CXC chemokine receptor 6 (CXCR6) is involved in tumor progression and the regulation of tumor immunity. However, its role in muscle-invasive bladder cancer (MIBC) remains largely unexplored.

**Methods:**

Data from 391 MIBC patients in the TCGA, 212 patients from GEO, 131 patients from our center, 195 patients in the IMvigor210 cohort, and single-cell RNA sequencing (scRNA-seq) data from 9 bladder cancer patients (GSE222315) were analyzed. Additionally, data from the GEPIA 2, TISCH2, TIMER2.0, and UALCAN platforms were utilized to investigate the prognostic and immunotherapeutic significance of CXCR6 in MIBC.

**Results:**

We observed that CXCR6 expression was significantly reduced in bladder cancer tumors and correlated with tumor stage and grade. Low CXCR6 expression was associated with poor recurrence-free survival (RFS) and overall survival (OS) in the TCGA cohort, a finding validated in both the meta-GEO dataset and our center’s cohort. Multivariate analysis confirmed that low CXCR6 expression was an independent predictor of poor OS and RFS. A nomogram incorporating CXCR6 expression and other independent prognostic factors was developed to accurately predict 3- and 5-year OS. Gene set enrichment analysis indicated that immune activation-related pathways were significantly enriched in tumors with high CXCR6 expression. CIBERSORT analysis revealed that CXCR6 expression was positively correlated with CD8+ T cells, CD4+ T cells, activated NK cells, M1 macrophages, and activated dendritic cells in TCGA, findings further validated by TIMER2.0. scRNA-seq data showed that CXCR6 was predominantly expressed in T and NK cells and facilitated T/NK-myeloid interaction via the CXCR6-CXCL16 axis. Importantly, CXCL16+ macrophages and dendritic cells recruited CXCR6+ T and NK cells, which exhibited enhanced cytotoxicity, thereby amplifying anti-tumor immunity. Clinically, in the IMvigor210 immunotherapy cohort, higher CXCR6 expression was associated with improved anti-PD-L1 therapeutic outcomes.

**Conclusion:**

Our findings highlight CXCR6 as a critical biomarker for predicting prognosis and immunotherapeutic response in MIBC.

## Introduction

Bladder cancer accounts for nearly 573,000 new cases globally each year, with muscle-invasive bladder cancer (MIBC) representing 25% of these cases (1, 2). Despite treatment with neoadjuvant cisplatin-based chemotherapy, approximately half of MIBC patients succumb to this aggressive disease within five years (3). While immunotherapies, such as immune checkpoint inhibitors (ICIs), show promise in advanced stage tumors, their effectiveness remains limited due to suboptimal response rates and significant adverse effects (4). Therefore, there is an urgent need to identify biomarkers that can predict ICI efficacy.

The role of the tumor microenvironment in tumor progression cannot be ignored (5–8). CXC chemokine receptor 6 (CXCR6), a member of the CXC chemokine family, plays an important role in tumor development (9). Its interaction with the ligand CXCL16 has been linked to cancer invasion and metastasis (10). Research indicates that CXCR6 expression can serve as a marker for T cell differentiation and may act as a cancer biomarker (11). CXCR6 is present on specific subsets of T cells and natural killer T (NKT) cells, while CXCL16 is predominantly found in dendritic cells, monocytes, and various tissue cells (12). The interaction between CXCR6 and CXCL16 guides immune cell homing, activation, expansion, and cytotoxic activity (13, 14). When CXCL16 binds to CXCR6, it triggers calcium mobilization and activates pathways such as ERK/MAPK and Akt/mTOR, along with downstream mediators like NF-κB, potentially leading to increased secretion of cytokines like tumor interferon-γ and necrosis factor-α (15). However, the clinical significance of CXCR6 in MIBC, and its potential as a predictive marker for immunotherapy efficacy in MIBC, remain unclear.

In this study, we evaluated CXCR6 expression and its prognostic significance in MIBC patients across different datasets. Using bulk and single-cell transcriptomic data, we found that tumors with high CXCR6 expression exhibited an immune-activated state. CXCR6 was predominantly expressed in T/NK cells and promoted T/NK-myeloid cells interaction via the CXCR6-CXCL16 axis. Importantly, we discovered that MIBC patients with high CXCR6 expression were more sensitive to anti-PD-L1 immunotherapy.

## Materials and methods

### Data acquisition

We obtained gene expression profiles and clinical data of MIBC patients from The Cancer Genome Atlas (TCGA) database, including 391 tumor samples, after excluding non-MIBC cases and those with incomplete data on age, recurrence-free survival (RFS), and overall survival (OS). Additional gene expression profiles and clinical data were retrieved from the Gene Expression Omnibus (GEO) database, focusing exclusively on MIBC patients. RNA-seq data were normalized to enhance comparability with microarray data. Three GEO datasets (GSE13507, n = 61; GSE31684, n = 78; GSE48075, n = 73) were merged to create a meta-GEO MIBC cohort (n = 212) for validating the prognostic value of CXCR6. For patients receiving anti-PD-L1 immunotherapy (IMvigor210), gene expression profiles and clinical data were obtained from the IMvigor210CoreBiologies repository.

### CXCR6 expression analysis

CXCR6 expression was analyzed using the UALCAN platform (https://ualcan.path.uab.edu) and GEPIA2 (http://gepia2.cancer-pku.cn/). These tools were utilized to compare CXCR6 expression between bladder cancer and normal tissues, as well as across different cancer stages.

### Survival analysis

Pan-cancer survival analysis for CXCR6 was conducted using the TIMER2.0 platform (http://timer.cistrome.org/). Additionally, Kaplan–Meier curves were used to assess survival times. To standardize the cutoff value for survival analysis in both the TCGA and meta-GEO cohorts, CXCR6 expression levels were normalized (mean = 0, SD = 1). The ‘‘minimum P value’’ method was applied to identify the optimal cutoff for CXCR6 expression, ensuring the best stratification of patients based on OS.

### Gene set enrichment analysis

GSEA was performed to investigate the potential mechanisms of CXCR6 in MIBC by identifying statistically significant gene sets between high and low CXCR6 expression groups. GSEA software v3.0 was used for the analysis, utilizing gene sets from the Molecular Signatures Database (MSigDB).

### Immune infiltration analysis

For immune microenvironment analysis, MIBC tissues were stratified into two groups based on CXCR6 expression levels. The CIBERSORT algorithm was applied to the TCGA dataset to assess the relationship between CXCR6 expression and 22 tumor-infiltrating immune cell subsets. Additionally, the correlation between CXCR6 expression and immune cell infiltration was further analyzed using TIMER2.0.

### scRNA-seq analysis

The scRNA-seq data from nine bladder cancer samples were downloaded from the GEO database under GSE222315 (16). Batch correction was performed using Harmony, and quality control along with clustering was conducted using Seurat, following standard preprocessing protocols (17). Principal component analysis (PCA) was used to reduce the dimensionality of the gene expression matrix, extracting the most essential features. Inter-cell distances were computed with the FindNeighbors function, which informed the construction of a shared-nearest neighbor graph. This was followed by clustering and cell grouping using the Louvain method. For data visualization, the PCA-reduced data was projected using UMAP (Uniform Manifold Approximation and Projection). To identify feature genes, the FindAllMarkers function was applied, and significant genes were determined using the Wilcoxon rank-sum test. Marker genes identified from the feature set were then utilized to assign cell type identities, referencing the CellMarker database (18) and relevant literature (19–24). Intercellular interactions were analyzed with CellChat, a tool for studying ligand-receptor signaling in specific pathways (25). To investigate the pathways and functions associated with CXCR6+ T/NK cells and CXCL16+ myeloid cells, gene enrichment analysis was conducted for each subcluster. Additionally, the scRNA-seq databases used to validate CXCR6 and CXCL16 expression were obtained from TISCH2 (http://tisch.comp-genomics.org).

### Patients in FUSCC cohort and immunohistochemistry

This study included 131 MIBC patients (pT2-T4, N0-N3, M0) who underwent radical cystectomy at Fudan University Shanghai Cancer Center (FUSCC) between 2008 and 2012. All patients provided informed consent and had not received neoadjuvant chemotherapy. Tumor tissue microarrays were constructed, and single-staining IHC was performed as previously described (26). The IHC score for CXCR6 was calculated by multiplying the staining intensity (0 = negative, 1 = weak, 2 = moderate, 3 = strong) by the percentage of positive cells (0-100%). Anti-CXCR6 antibody (1:300, Proteintech, #36380) was used for staining, with a median IHC score of 180 serving as the cutoff. CXCR6 expression levels below 180 were classified as low expression. Two pathologists independently reviewed the slides, and all tissue microarray samples were stained concurrently.

### Statistical analyses

Pearson’s χ² test was used to compare categorical variables, while the t-test was employed for continuous variables. For nonparametric data, the Mann-Whitney U test (Wilcoxon Rank Sum Test) was applied to compare outcomes between two independent groups. Survival curves were analyzed using the Kaplan-Meier method and compared with log-rank tests. Univariate and multivariate analyses were performed using the Cox regression model, with variables significant in the univariate analysis included in the multivariate model. A prognostic nomogram and calibration plots were created using R software (v3.0.2) with the ‘rms’ package. All statistical analyses were carried out using SPSS v21.0 (IBM Corp), and a two-sided P-value <0.05 was considered statistically significant.

## Results

### CXCR6 expression predicts favorable outcomes in MIBC patients

**Figure 1:**
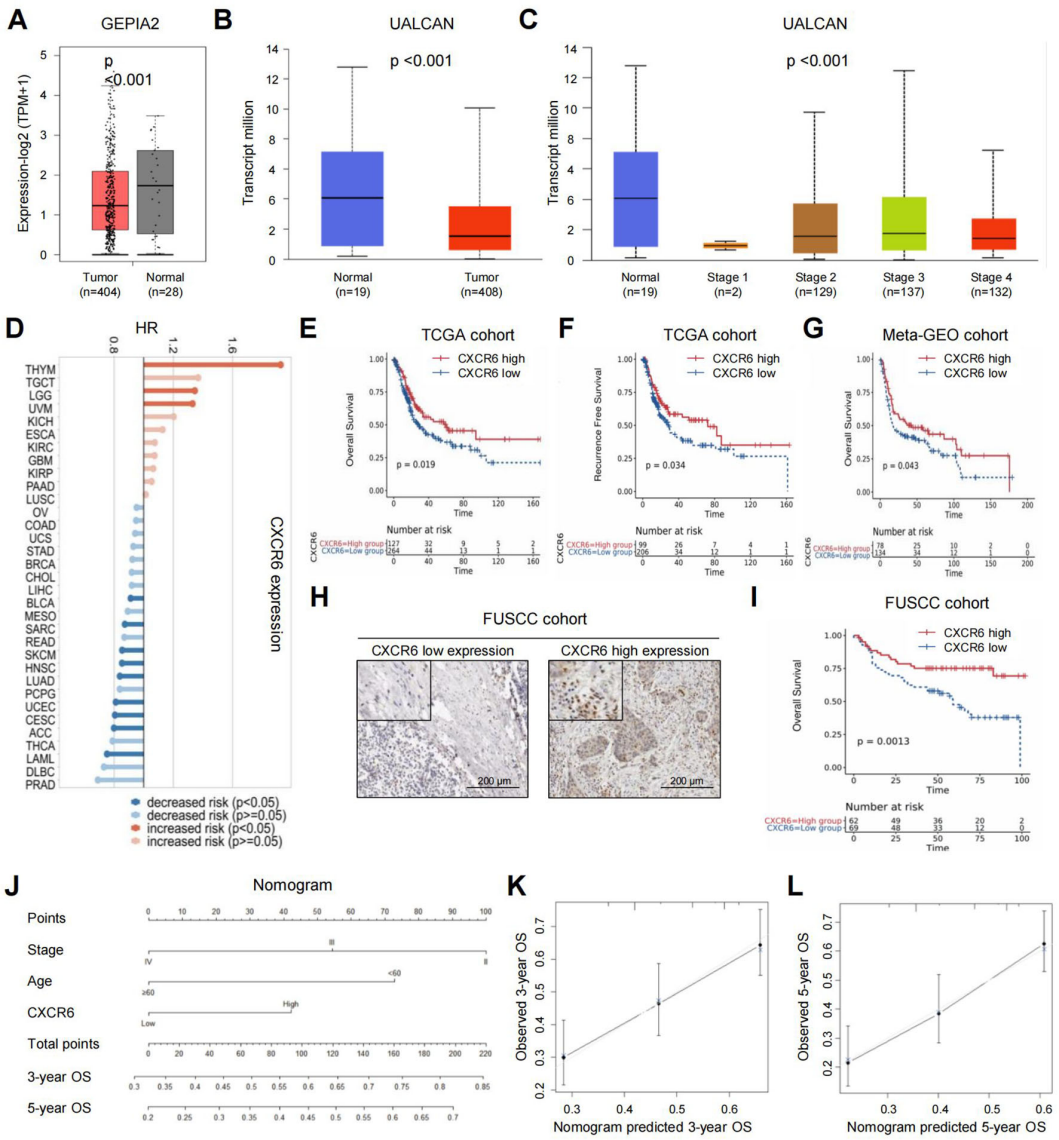
High CXCR6 expression predicts favorable outcomes in MIBC patients. **(A)** CXCR6 expression in bladder cancer tissues versus normal tissues using GEPIA2. **(B)** CXCR6 expression in bladder cancer tissues versus normal tissues using UALCAN. **(C)** CXCR6 expression in different stages of bladder cancer using UALCAN. **(D)** Forest plot of OS prognostic analysis for CXCR6 in pan-cancer using TISCH2. **(E–G)** Kaplan–Meier survival curves showing the association between CXCR6 expression and patient outcomes in the TCGA database **(E, F)** and meta-GEO database **(G)**. **(H)** Representative images of high and low CXCR6 expression in the FUSCC cohort. **(I)** Kaplan–Meier survival curves showing the association between CXCR6 expression and OS in the FUSCC cohort. **(J)** Nomogram predicting the 3- and 5-year OS for MIBC patients after surgery. **(K, L)** Calibration plots for 3-year **(K)** and 5-year **(L)** OS predictions following surgery.

First, we investigated the expression of CXCR6 in bladder cancer. Using the GEPIA2 and UALCAN online platforms, we found that CXCR6 expression was significantly lower in bladder cancer tumor tissues compared to adjacent normal tissues (**Figures 1A, B**). Moreover, CXCR6 expression decreased with advancing tumor stage (**Figure 1C**).

Next, we explored the prognostic significance of CXCR6 in MIBC. A forest plot of overall survival (OS) prognostic analysis across different cancer types using TISCH2 revealed that CXCR6’s predictive value varied among cancers (**Figure 1D**). Specifically, in bladder cancer, low CXCR6 expression was associated with worse patient outcomes. In particular, we assessed the prognostic value of CXCR6 in MIBC using the TCGA dataset. Applying the “minimum P value” approach to define the cutoff, 264 patients (67.5%) were classified as having low CXCR6 expression, while 127 patients (32.5%) had high expression. The characteristics of patients in the high and low CXCR6 expression groups were summarized in **Supplementary Table 1**. Low CXCR6 expression was significantly associated with higher tumor grade. Kaplan–Meier analysis further demonstrated that low CXCR6 expression was significantly correlated with worse OS and RFS in the TCGA cohort (**Figures 1E, F**). This finding was validated in the meta-GEO database, where patients with high CXCR6 expression showed a trend toward better OS compared to those with low expression (**Figure 1G**). Additionally, IHC analysis in a 131-patient FUSCC cohort further confirmed that low CXCR6 expression was associated with poorer OS (**Figures 1H, I**). Cox regression analyses were performed to evaluate whether CXCR6 expression was an independent predictor of survival outcomes. After adjusting for confounding factors such as age, gender, tumor weight, stage, and necrosis, multivariate analysis showed that CXCR6 expression was an independent prognostic factor for both OS and RFS (**Supplementary Table 1**).

To provide a practical tool for prognosis, we developed a nomogram to predict 3- and 5-year OS following surgery (**Figure 1J**). The model incorporated age at surgery, tumor stage, and CXCR6 expression as key predictors, with higher total scores indicating worse prognosis. Calibration plots showed that the nomogram accurately predicted 3- and 5-year survival, closely aligning with the ideal model (**Figures 1K, L**). In summary, high CXCR6 expression is a favorable prognostic marker in MIBC patients.

### High CXCR6 expression is associated with an immune-activated tumor microenvironment in MIBC

**Figure 2:**
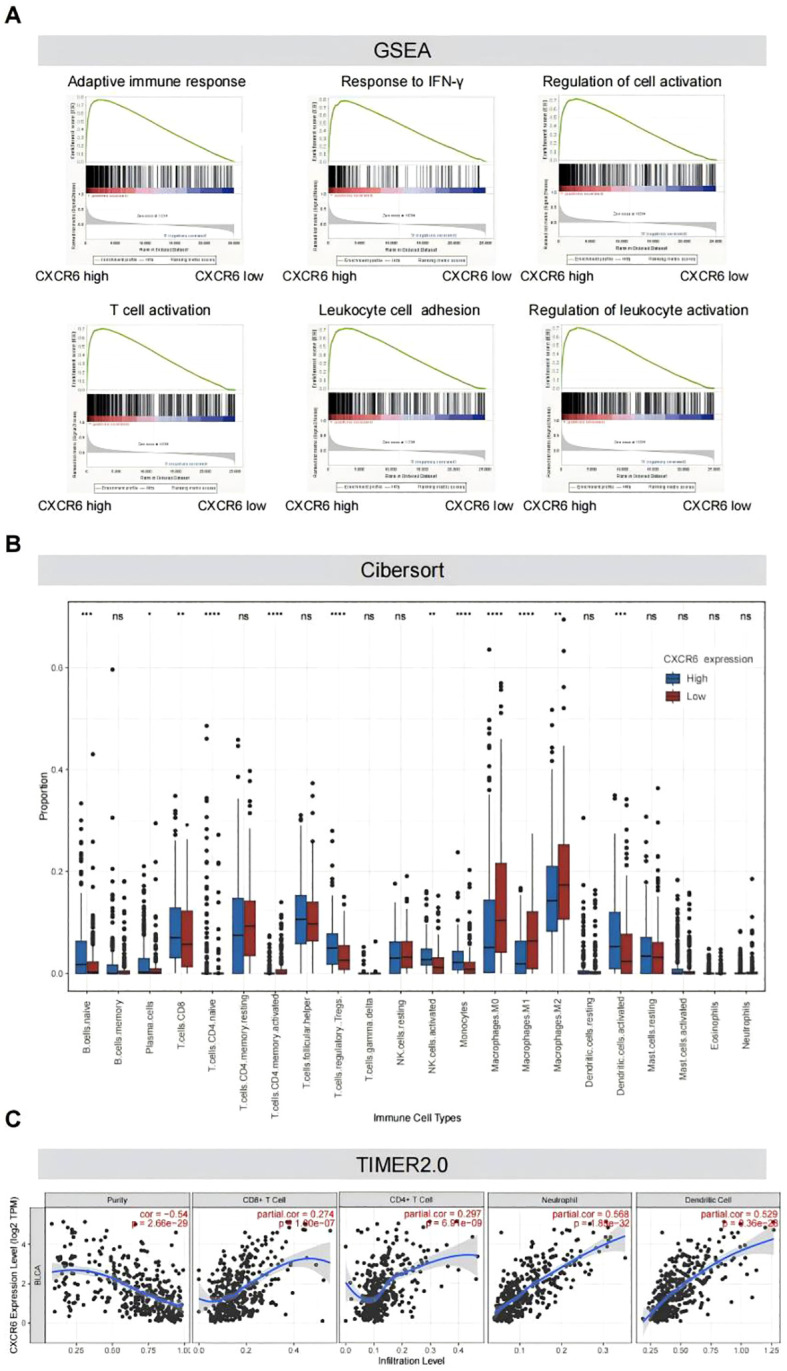
High CXCR6 expression is associated with an immune-activated tumor microenvironment in MIBC. **(A)** GSEA plots demonstrating significant enrichment of pathways in high-CXCR6 MIBC tumors from the TCGA dataset. **(B)** Immune infiltration levels in high-CXCR6 and low-CXCR6 groups analyzed by CIBERSORT. ns indicates no significance; * indicates P < 0.05; ** indicates P < 0.01; *** indicates P < 0.001; **** indicates P < 0.0001. **(C)** Correlation analysis between CXCR6 expression and immune infiltration performed using TIMER2.0.

To further explore the molecular mechanisms underlying survival outcomes, we conducted a biological process and pathway analysis of MIBC from the TCGA dataset using GSEA. The results revealed that immune activation-related pathways were significantly enriched in tumors with high CXCR6 expression, including adaptive immune response, interferon gamma response, cell activation, T cell activation, leukocyte adhesion, and lymphocyte activation (**Figure 2A**; **Supplementary Table 2**). These findings are consistent with previous studies that emphasize the critical role of CXCR6 in regulating tumor immunity.

To further investigate the correlation between CXCR6 expression and immune cell infiltration, we utilized CIBERSORT to analyze CXCR6 expression in 391 MIBC samples and assess the proportions of 22 immune cell subtypes. The analysis revealed that several immune cell types, including naïve B cells, plasma cells, naïve CD4+ T cells, activated CD4+ memory T cells, activated NK cells, monocytes, macrophages (M0, M1, M2), and activated dendritic cells (DCs), were significantly less abundant in the low CXCR6 expression group compared to the high expression group (**Figure 2B**). To further validate the correlation between CXCR6 expression and immune cell infiltration, we used TIMER2.0. Consistent with previous findings, CXCR6 expression was significantly positively correlated with CD8+ T cells, CD4+ T cells, neutrophils, and dendritic cells (DC) (**Figure 2C**). These results suggest that CXCR6 plays a key role in promoting immune activation in MIBC.

### scRNA-seq identifies CXCR6 in T/NK cells mediating T/NK-myeloid interaction via CXCL16

**Figure 3:**
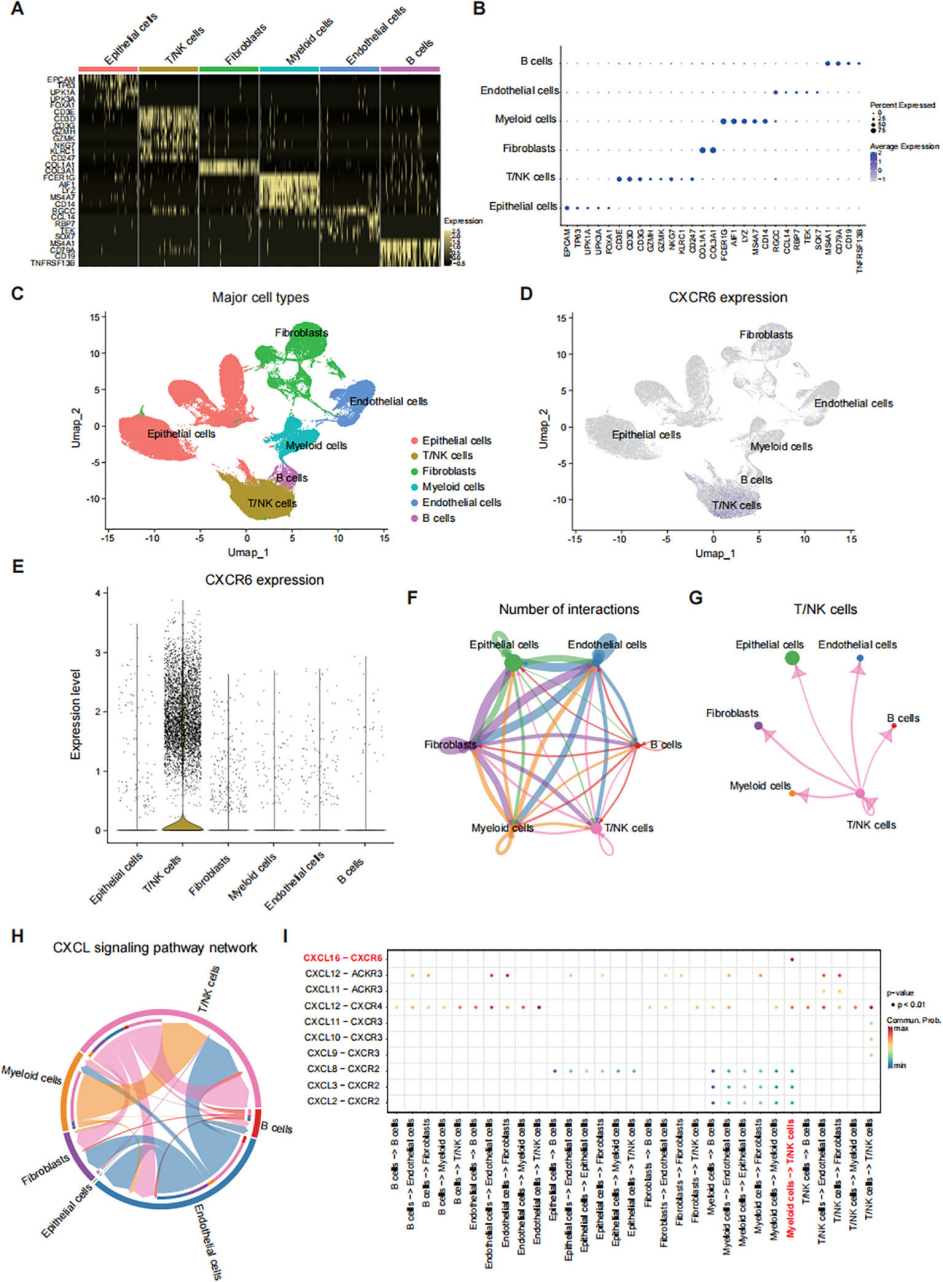
scRNA-seq identifies CXCR6 in T/NK cells mediating T/NK-myeloid interaction via CXCL16. **(A)** Heatmap showing the expression levels of major cell type markers in bladder cancer tissues from 9 patients (GSE222315). **(B)** Dot plot displaying the distribution of expression levels for key cell type markers. **(C)** UMAP plot of 76,328 cells post-quality control and filtering, grouped by major cell types. **(D)** UMAP plot depicting CXCR6 expression across different major cell types. **(E)** Violin plot illustrating CXCR6 expression levels across various cell types. **(F)** Cell-cell interaction network among major cell types. **(G)** Interaction network between T/NK cells and other major cell types. **(H)** Chord diagram representing the CXCL signaling network among different cell types. **(I)** Summary of selected ligand-receptor interactions between major cell types in the CXCL signaling pathway.

To explore the role of CXCR6 in cellular composition and molecular profiles, we analyzed scRNA-seq data from 9 bladder cancer samples (GSE222315) (16). After integrating the transcriptional data, we identified six major cell types based on known cell-type-specific markers: epithelial cells, T/NK cells, fibroblasts, myeloid cells, endothelial cells, and B cells (**Figures 3A–C**). Notably, T/NK cells exhibited the highest levels of CXCR6 expression among these clusters (**Figures 3D, E**). Similar results were obtained from the public single-cell bladder cancer dataset (GSE14952) analyzed using TISCH2, further confirming the pivotal role of this receptor in these immune subsets (**Supplementary Figure 1A**).

Additionally, cell communication analysis using receptor-ligand interaction networks revealed that T/NK cells were connected with various cell types, including myeloid cells (**Figures 3F, G**). A closer examination indicated that T/NK-myeloid interaction was primarily mediated by the CXCL16-CXCR6 axis (**Figures 3H, I**). Notably, myeloid cells displayed the highest levels of CXCL16 expression (**Supplementary Figure 1B**), suggesting that CXCR6+ T/NK cells are actively recruited and influenced by CXCL16+ myeloid cells. This was further supported by similar findings from the TISCH2 analysis of the GSE145281 dataset (**Supplementary Figure 1C**). These findings suggest that CXCR6 is predominantly expressed in T/NK cells and plays a critical role in promoting T/NK-myeloid interaction through the CXCL16-CXCR6 axis.

### Myeloid cells enhance T and NK cell cytotoxicity via the CXCL16-CXCR6 axis

**Figure 4:**
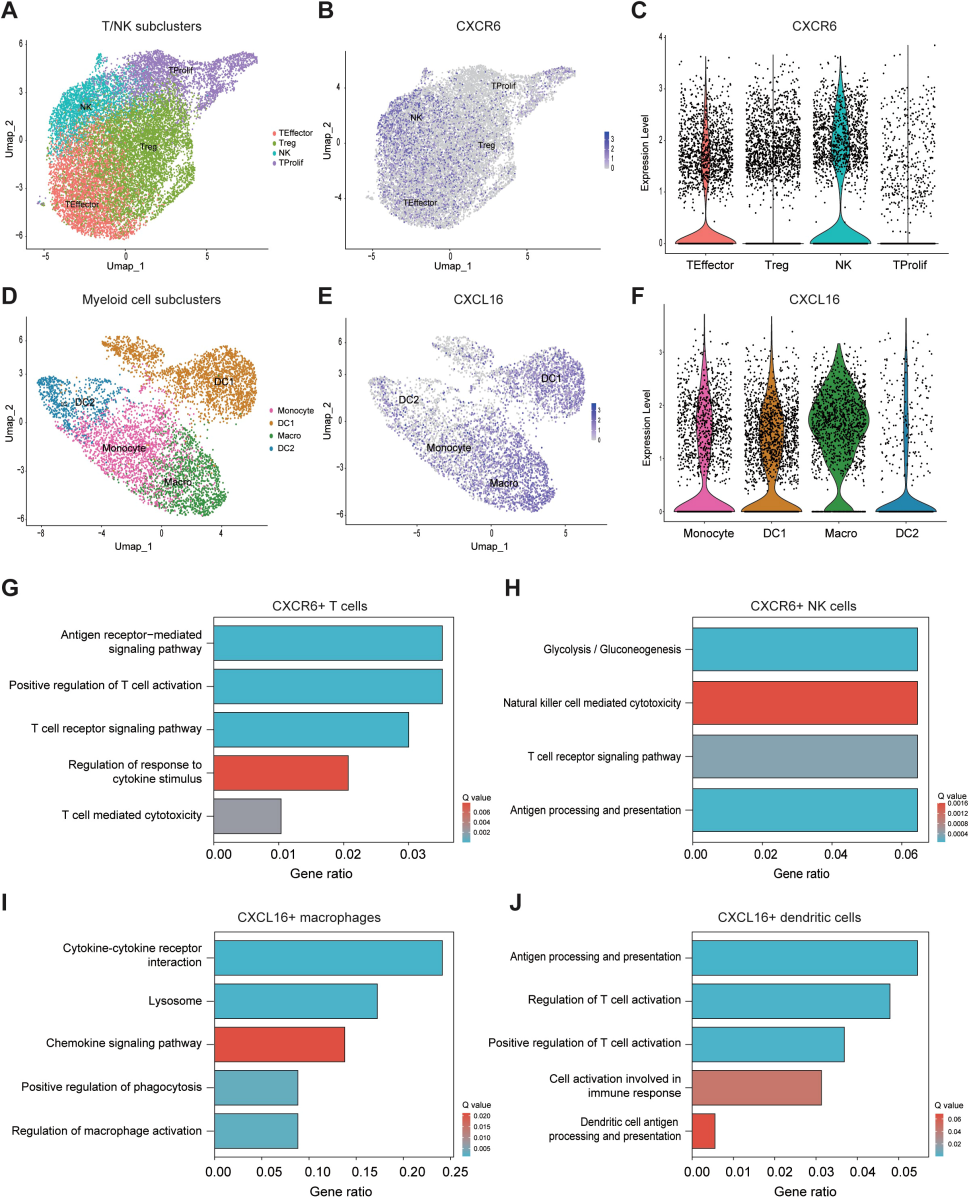
Myeloid cells enhance T and NK cell cytotoxicity via the CXCL16-CXCR6 axis. **(A)** UMAP plot showing subclusters of T/NK cells. **(B)** UMAP plot illustrating CXCR6 expression across various T/NK cell subclusters. **(C)** Violin plot comparing CXCR6 expression levels among different T/NK cell subclusters. **(D)** UMAP plot showing subclusters of myeloid cells. **(E)** Violin plot comparing CXCR6 expression levels across different myeloid cell subclusters. **(F)** Violin plot illustrating CXCR6 expression levels across different myeloid cell subclusters. **(G–J)** Pathway enrichment analysis of CXCR6+ T cells **(G)**, CXCR6+ NK cells **(H)**, CXCL16+ macrophages **(I)**, and CXCL16+ DCs **(J)**.

Next, we further divided T/NK cells into four subclusters: T effector, T regulatory (T reg), T proliferative, and NK cells (**Figure 4A**; **Supplementary Figure 2A**). We found that CXCR6 was predominantly expressed in T effector cells and NK cells (**Figures 4B, C**). Similarly, we subdivided myeloid cells into four subclusters: monocytes, macrophages, and DC1 and DC2 (**Figure 4D**; **Supplementary Figure 2B**). CXCL16 was mainly expressed in macrophages and DC1 cells (**Figures 4E, F**).

Subsequently, we performed pathway enrichment analysis on CXCR6+ T cell, CXCR6+ NK cells, CXCL16+ macrophages, and CXCL16+ DC1 cells to further elucidate the functional roles of these cell subclusters. The results showed that CXCR6+ T cells were significantly enriched in pathways related to antigen receptor-mediated signaling, positive regulation of T cell activation, T cell receptor signaling, regulation of response to cytokine stimulus, and T cell-mediated cytotoxicity (**Figure 4G**). For CXCR6+ NK cells, the analysis highlighted the enrichment of pathways involved in natural killer cell-mediated cytotoxicity, T cell receptor signaling, and antigen processing and presentation (**Figure 4H**). In CXCL16+ macrophages, pathways related to cytokine-cytokine receptor interaction, chemokine signaling, positive regulation of phagocytosis, and regulation of macrophage activation were significantly enriched (**Figure 4I**). Finally, CXCL16+ DC1 cells were enriched in pathways related to antigen processing and presentation, regulation of T cell activation, positive regulation of T cell activation, cell activation involved in immune response, and dendritic cell antigen processing and presentation (**Figure 4J**). Taken together, these findings suggest that the CXCL16-CXCR6 axis mediates important immune interactions between T/NK cells and myeloid cells, particularly macrophages and DC1 cells, which are essential for orchestrating a robust anti-tumor immune response (27–30).

### CXCR6 expression predicts the benefit of immunotherapy in MIBC

**Figure 5:**
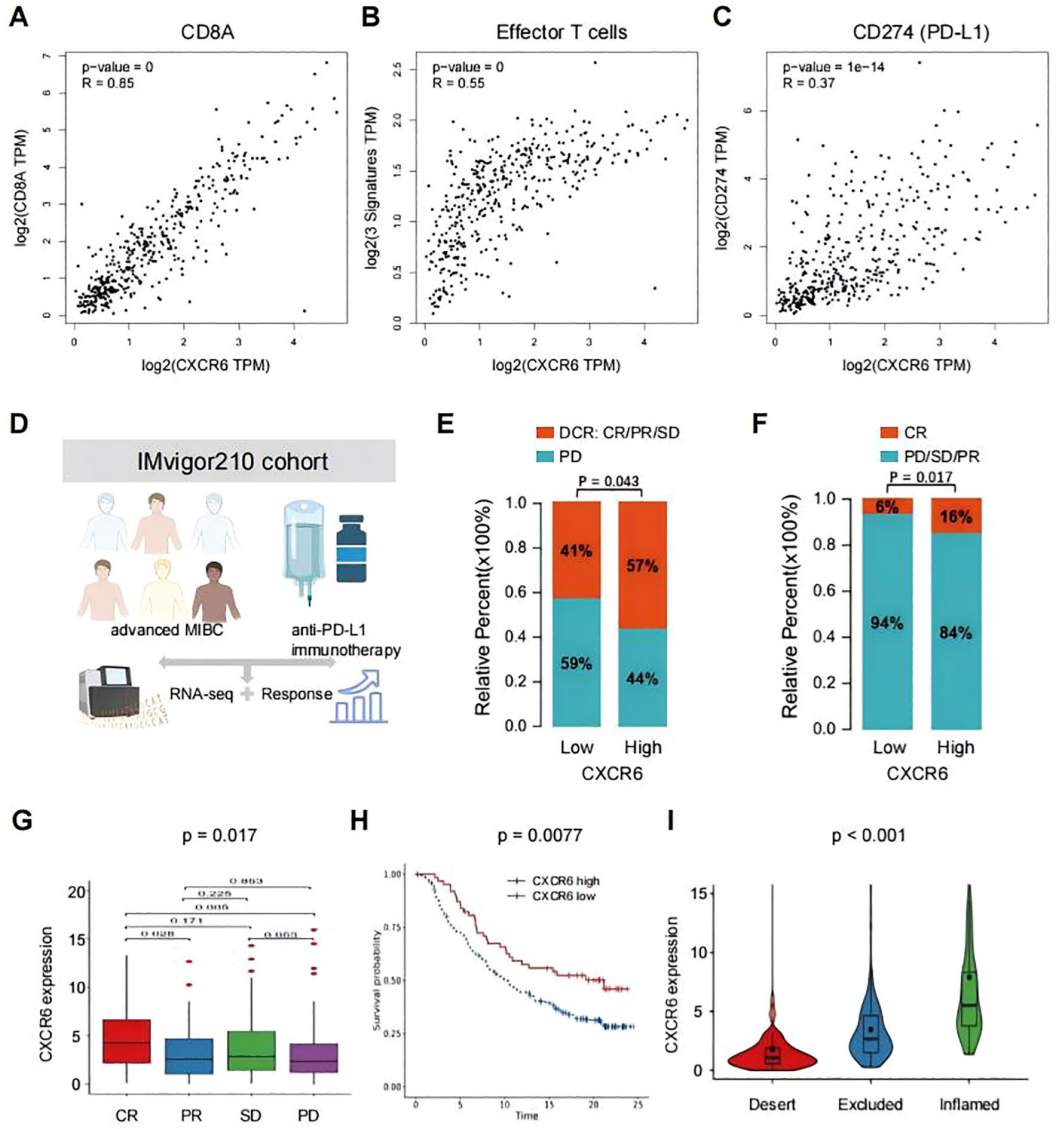
CXCR6 expression predicts the benefit of immunotherapy in bladder cancer. **(A–C)** Correlation between CXCR6 expression and immunotherapy benefit markers, including CD8A **(A)**, effector T cell signature **(B)**, and CD274 (PD-L1) **(C)**, in bladder cancer, analyzed using GEPIA2. **(D)** Overview of the IMvigor210 clinical trial. **(E, F)** Comparison of immunotherapy outcomes in patients with high or low CXCR6 expression receiving anti-PD-L1 treatment. DCR, disease control rate; CR, complete response; PR, partial response; SD, stable disease; PD, progressive disease. **(G)** CXCR6 expression levels across different anti-PD-L1 clinical response groups. **(H)** Kaplan-Meier survival analysis of patients with high or low CXCR6 expression receiving anti-PD-L1 immunotherapy. **(I)** CXCR6 expression levels among distinct tumor immune phenotypes.

Given the earlier findings that CXCR6 promoted anti-tumor immunity in MIBC, we investigated its potential role in predicting immunotherapy response. First, we conducted a correlation analysis between CXCR6 and previously reported immunotherapy biomarkers using GEPIA2. The results revealed a significant positive correlation between CXCR6 expression and CD8A (**Figure 5A**), effector T cell signature (**Figure 5B**), and CD274 (PD-L1) (**Figure 5C**) in bladder cancer, suggesting that CXCR6 may hold potential as a predictive biomarker for immunotherapy response.

To further investigate the association between CXCR6 and immunotherapy outcomes, we analyzed the IMvigor210 cohort, a well-established phase II clinical trial evaluating the efficacy of anti-PD-L1 (atezolizumab) immunotherapy in advanced MIBC patients (31, 32) (**Figure 5D**). Our analysis revealed that patients with high CXCR6 expression demonstrated greater disease control (**Figure 5E**) and higher complete response rates (**Figure 5F**) compared to those with low CXCR6 expression. Additionally, lower CXCR6 expression was associated with poorer treatment responses (**Figure 5G**), whereas higher CXCR6 expression correlated with improved overall survival (OS) following immunotherapy (**Figure 5H**). The immune phenotypes of tumors in the IMvigor210 cohort have been detected, so we investigated the associations of CXCR6 expression among different immune phenotypes. We found that high CXCR6 expression was significantly associated with the inflamed immune phenotype (**Figure 5I**), which is known to respond more favorably to immunotherapy, reinforcing CXCR6 as a potential biomarker for predicting immunotherapy efficacy.

## Discussion

In this study, we identified CXCR6 as a critical player in the progression and immune regulation of MIBC. Our comprehensive analysis revealed that low CXCR6 expression was associated with poor survival outcomes and reduced immune cell infiltration. Importantly, we demonstrated that CXCR6 was predominantly expressed in T and NK cells and played a pivotal role in mediating anti-tumor immunity through interactions with CXCL16+ macrophages and DC. These findings suggest that CXCR6 may serve as both a prognostic biomarker and a predictive marker for immunotherapeutic response, highlighting its potential as a novel therapeutic target in MIBC.

Increased CXCR6 expression has also been observed across several cancers, including renal, prostate, breast, and liver cancers, where it served as a prognostic marker for overall survival (33–36). Consistent with these findings, we identified CXCR6 as an independent prognostic biomarker for both OS and RFS in MIBC based on analyses of public datasets and the FUSCC cohort. These results stand in contrast to a study by Lee et al., which found no significant correlation between CXCR6 expression and bladder cancer outcomes (37). Our study, focused specifically on MIBC, provided stronger evidence of CXCR6’s prognostic value, though external validation is still needed. Given the molecular diversity of MIBC, traditional clinicopathological features do not fully predict patient prognosis (38). Thus, easily measurable biomarkers, such as CXCR6, are urgently needed to guide post-surgical follow-up and facilitate personalized treatment plans. Our study found that CXCR6 expression was significantly correlated with clinical outcomes in MIBC patients after surgery. Additionally, the prognostic nomogram we developed—integrating patient age, AJCC stage, and CXCR6 expression—outperformed traditional models in predicting OS. Nevertheless, further prospective studies are necessary to validate the prognostic utility of CXCR6 in clinical practice.

Initially identified as a co-receptor for HIV on human memory T cells and NK cells (39, 40), CXCR6 has more recently been recognized for its involvement in tumor immunity (41). Notably, CXCR6 is crucial for the long-term tumor control mediated by CD8+ cytotoxic T lymphocytes within the tumor microenvironment (11). CXCR6+ CD8+ T cells are frequently found in tumors and are associated with improved survival outcomes in cancer patients (12), as well as enhanced antitumor responses and better efficacy of checkpoint blockade therapies (14). Hornburg et al. further demonstrated that tumor-intrinsic features and chemokine interactions—particularly the CXCL16-CXCR6 axis—shaped immune cell infiltration patterns in ovarian tumors (42). Additionally, CXCR6 serves as a marker for resident memory T (TRM) cells, which play a role in immunosurveillance through interactions with epithelial cells (43, 44). Targeting and expanding antigen-specific TRM expressing CXCR6 could offer new therapeutic avenues, with its potential as a biomarker of response to immunotherapy opening promising perspectives in MIBC treatment.

The success of immunotherapy is increasingly understood to be partly due to the immune landscape of the tumor microenvironment (45). Tumors with an immune-inflamed phenotype, or “hot tumors,” tend to respond more favorably to ICIs, whereas “cold tumors” generally exhibit resistance to such therapies (46). In light of this, we investigated how CXCR6 shaped the immunogenic features of the tumor microenvironment in MIBC. Our findings revealed that CXCR6 expression correlated positively with several immune cell infiltrates, particularly T cells, NK cells, macrophages, and DC. Furthermore, anti-tumor immunity-related pathways were significantly enriched in the CXCR6 high-expression group, suggesting that CXCR6 upregulation enhanced immune surveillance and suppressed tumor progression. scRNA-seq confirmed that CXCR6 was predominantly expressed in CD8+ T cells and NK cells, enhancing their cytotoxic functions. Moreover, macrophages and DC1 cells within the tumor microenvironment secreted CXCL16, which recruited CXCR6+ CD8+ T cells and CXCR6+ NK cells to bolster anti-tumor responses. This coordinated immune response, mediated by the CXCL16-CXCR6 axis, suggests that targeting this pathway could be a promising therapeutic approach to enhance immune responses in MIBC.

This study has two main limitations. First, its retrospective design and lack of external validation limited the generalizability of our findings. While we demonstrated the prognostic value of CXCR6 in predicting clinical outcomes and responses to ICIs, prospective studies and external validation are essential before CXCR6 can be implemented in routine clinical practice. Second, further functional studies are required to elucidate the specific mechanisms by which CXCR6 regulates MIBC immunology, which will provide deeper insights into its potential as a therapeutic target.

In conclusion, our study highlights the potential of CXCR6 as a prognostic biomarker and therapeutic target in MIBC. CXCR6 expression correlates with enhanced immune surveillance and anti-tumor responses, particularly through its role in the CXCL16-CXCR6 axis. While these findings broaden our understanding of CXCR6’s role in the tumor microenvironment, further studies are required to validate its clinical utility and to elucidate its detailed mechanisms in MIBC progression. Ultimately, these insights may contribute to the development of more precise, immune-based therapeutic strategies for MIBC patients.

## Data Availability

The data presented in the study can be found in online repositories. The names of the repositories and accession numbers can be found in the article/**Supplementary Material**. Further inquiries can be directed to the corresponding author.
