# Supplementary material for: CXCR6 expression predicts prognosis and immunotherapeutic benefit in muscle-invasive bladder cancer
Source: Front Oncol. 2024 Nov 11;14:1498579. doi: 10.3389/fonc.2024.1498579 (PMC11586233; doi:10.3389/fonc.2024.1498579)
Supplement: Supplementary file 1 [file DataSheet1.docx]

**Supplementary for**

**CXCR6 expression predicts prognosis and immunotherapeutic benefit in muscle-invasive bladder cancer**

**Supplementary includes:**

1. **Supplementary Tables**
2. **Supplementary Figures 1-2**

**Supplementary Table 1. Patient characteristics and associations with expression of CXCR6.**

|  | | **TCGA cohort ( *n* = 391 )** | | | | | |
| --- | --- | --- | --- | --- | --- | --- | --- |
| **Characteristics** | | **Patients** | | **CXCR6** | |  | |
|  |  | **No.** | **%** | **Low** | **High** | ***P**** | |
| **Total** | | 391 | 100 | 264 (67.5%) | 127 (32.5%) |  | |
| **Age at surgery(year)** | |  |  |  |  | 0.510 | |
|  | Median (IQR) | 68 (60-76) | | 69 (60-77) | 68 (60-75) |  | |
| **Gender** | |  |  |  |  | 0.183 | |
|  | Male | 285 | 72.9 | 198 (16.9%) | 87 (10.2%) |  | |
|  | Female | 106 | 27.1 | 66 (50.6%) | 40 (22.3%) |  | |
| **Tumor weight (g)** | |  |  |  |  | 0.887 | |
|  | Median (IQR) | 239 (180-369.5) | | 243 (176.25-372.75) | 225 (186-369.50) |  | |
| **AJCC stage** | |  |  |  |  | 0.192 | |
|  | II | 125 | 32 | 81 (20.7%) | 44 (11.3%) |  | |
|  | III | 137 | 35 | 88 (22.5%) | 49 (12.5%) |  | |
|  | IV | 129 | 33 | 95 (24.3%) | 34 (8.70%) |  | |
| **Grade** | |  |  |  |  | **0.024** | |
|  | Low grade | 20 | 5.1 | 19 (4.9%) | 1 (0.3%) |  | |
|  | High grade | 369 | 94.4 | 244 (62.4%) | 125 (32.0%) |  | |
| **Events** | |  |  |  |  |  | |
|  | Death | 173 | 44.2 | 122 (31.2%) | 51 (13%) | 0.278 | |
|  | Recurrence | 262 | 67 | 169 (43.2%) | 93 (23.8%) | 0.085 | |
| IQR: interquartile range; AJCC: American Joint Committee on Cancer. | | | | | | |  |
| *P** value from Fisher’s exact test was used when data fail to meet the requirement of Chi-square test. | | | | | | |  |
|  | | | | | | |  |

| **Supplementary Table 2.** **Multivariate cox regression analyses for recurrence-free survival and overall survival.** | | |
| --- | --- | --- |
| **Characteristics** | **TCGA Cohort ( *n* = 391 )** | |
|  | **HR (95%CI)** | ***P**** |
| **Overall survival** |  |  |
| Age (≥60y vs. <60y) | 1.841 (1.231,2.753) | **0.003** |
| Gender (male vs. female) | 0.856 (0.606,1.208) | 0.376 |
| Tumor weight (≥200g vs. <200g) | 1.048 (0.742,1.481) | 0.790 |
| Necrosis (≥10% vs. <10%) | 1.192 (0.853,1.665) | 0.303 |
| Stage (III+IV vs. II) | 1.972 (1.340,2.904) | **0.001** |
| CXCR6 (high vs. low) | 0.662 (0.469,0.934) | **0.019** |
| **Recurrence-free survival** |  |  |
| Age (≥60y vs. <60y) | 1.372 (0.925,2.035) | 0.116 |
| Gender (male vs. female) | 0.835 (0.563,1.236) | 0.367 |
| Tumor weight (≥200g vs. <200g) | 1.284 (0.871,1.894) | 0.207 |
| Necrosis (≥10% vs. <10%) | 1.396 (0.962,2.025) | 0.079 |
| Stage (III+IV vs. II) | 2.283 (1.490,3.498) | **<0.001** |
| CXCR6 (high vs. low) | 0.626 (0.424,0.924) | **0.018** |
| HR = hazard ratio; CI = confidence interval. | | |
| *P** value < 0.05 shows statistically significant. | | |

**Supplementary Table 3. Gene sets enriched in phenotypes.**

| **Gene set name** | **NES** | **NOM p-val** | **FDR q-val** |
| --- | --- | --- | --- |
| **CXCR6 high expression** | |  | |
| GO_ADAPTIVE_IMMUNE_RESPONSE | 3.35 | <0.001 | <0.001 |
| GO_RESPONSE_TO_INTERFERON_GAMMA | 3.24 | <0.001 | <0.001 |
| GO_POSITIVE_REGULATION_OF_CELL_ACTIVATION | 3.16 | <0.001 | <0.001 |
| GO_T_CELL_ACTIVATION | 3.14 | <0.001 | <0.001 |
| GO_LEUKOCYTE_CELL_CELL_ADHESION | 3.12 | <0.001 | <0.001 |
| GO_REGULATION_OF_LYMPHOCYTE_ACTIVATION | 3.12 | <0.001 | <0.001 |
| **CXCR6 low expression** | |  | |
| GO_MONOCARBOXYLIC_ACID_CATABOLIC_PROCESS | -2.25 | <0.001 | 0.002 |
| GO_FATTY_ACID_BETA_OXIDATION | -2.21 | <0.001 | 0.002 |
| GO_FATTY_ACID_CATABOLIC_PROCESS | -2.13 | <0.001 | 0.008 |
| GO_GPI_ANCHOR_METABOLIC_PROCESS | -1.97 | <0.001 | 0.076 |
| GO_PREASSEMBLY_OF_GPI_ANCHOR_IN_ER_MEMBRANE | -1.97 | <0.001 | 0.064 |
| GO_DRUG_CATABOLIC_PROCESS | -1.96 | <0.001 | 0.062 |

NES: normalized enrichment score; NOM: nominal; FDR: false discovery rate.

Gene sets with NOM p-value < 0.05 and FDR q-value < 0.05 are considered as significant.

**Supplementary figures and figure legends**


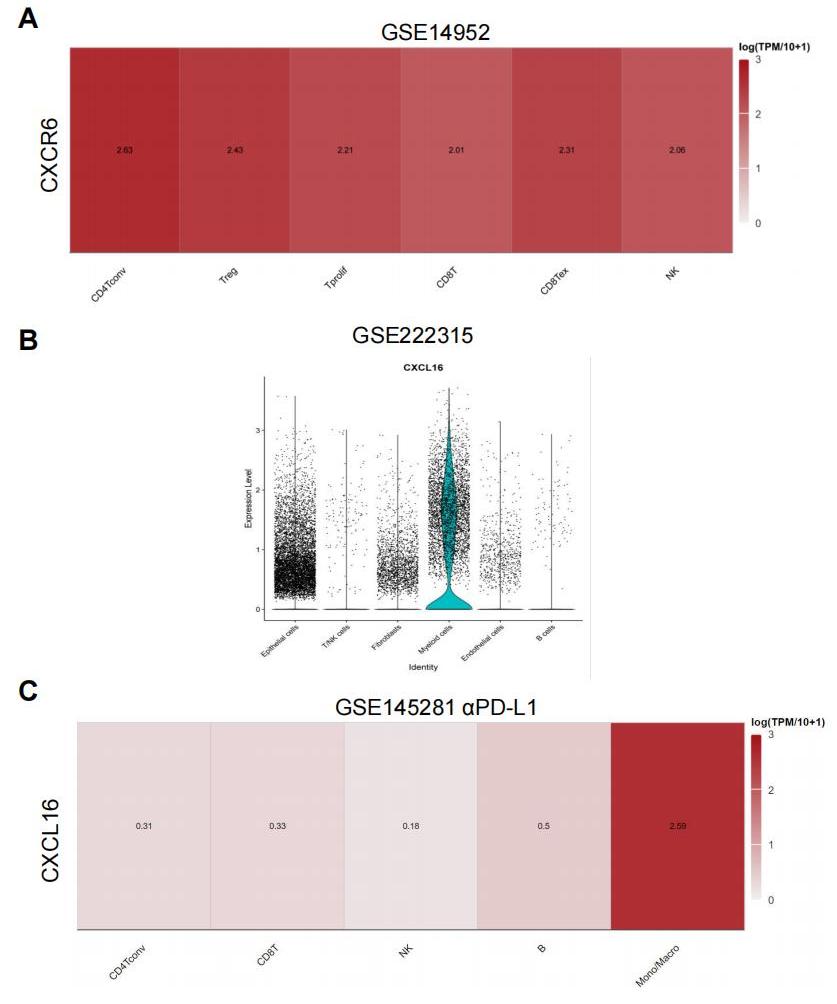


**Supplementary Figure 1. Analysis of CXCR6 and CXCL16 expression in scRNA-seq.**

**(A)** CXCR6 expression across cell clusters in the GSE14952 scRNA-seq data from TISCH2.

**(B)** CXCL16 expression across cell clusters in the GSE222315 scRNA-seq data.

**(C)** CXCL16 expression across cell clusters in the GSE145281 scRNA-seq data from TISCH2.


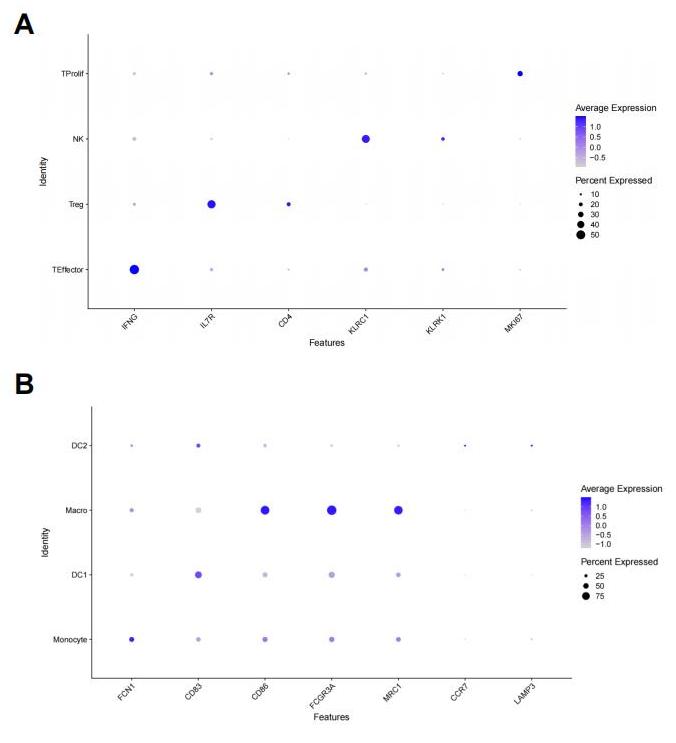


**Supplementary Figure 2. Key cell type markers of T/NK cells and myeloid cells.**

**(A, B)** Dot plot displaying the distribution of expression levels for key cell type markers of T/NK cells (A) and myeloid cells (B).
